# Strategies for Early Prediction and Timely Recognition of Drug-Induced Liver Injury: The Case of Cyclin-Dependent Kinase 4/6 Inhibitors

**DOI:** 10.3389/fphar.2019.01235

**Published:** 2019-10-24

**Authors:** Emanuel Raschi, Fabrizio De Ponti

**Affiliations:** Pharmacology Unit, Department of Medical and Surgical Sciences, Alma Mater Studiorum, University of Bologna, Bologna, Italy

**Keywords:** drug-induced liver injury, hepatotoxicity, predictivity, signal detection, risk ranking

## Abstract

The idiosyncratic nature of drug-induced liver injury (DILI) represents a current challenge for drug developers, regulators and clinicians. The myriad of agents (including medications, herbals, and dietary supplements) with recognized DILI potential not only strengthens the importance of the post-marketing phase, when urgent withdrawal sometimes occurs for rare unanticipated liver toxicity, but also shows the imperfect predictivity of pre-clinical models and the lack of validated biomarkers beyond traditional, non-specific liver function tests. After briefly reviewing proposed key mechanisms of DILI, we will focus on drug-related risk factors (physiochemical and pharmacokinetic properties) recently proposed as predictors of DILI and use cyclin-dependent kinase 4/6 inhibitors, relatively novel oral anticancer medications approved for breast cancer, as a case study to discuss the feasibility of early detection of DILI signals during drug development: published data from pivotal clinical trials, unpublished post-marketing reports of liver adverse events, and pharmacokinetic properties will be used to provide a comparative evaluation of their liver safety and gain insight into drug-related risk factors likely to explain the observed differences.

## Introduction

Drug-induced liver injury (DILI) is a public health issue of utmost interest for drug companies, regulatory agencies, and hepatologists: though from different perspectives, all these stakeholders are concerned with early and timely identification of liver damage both during drug development and in clinical practice.

The most challenging form of DILI is the so-called idiosyncratic one, because, by definition, it is unpredictable: it is usually unrelated to the dose (although some degree of dose dependency exists (Roth and Ganey, 2010), occurs only in a small fraction of subjects exposed, and is characterized by a variable onset time (with delayed latency in different cases), with a number of phenotypes, which further complicate recognition and differential diagnosis.

This scenario reflects our partial and superficial mechanistic understanding, with still imperfect predictivity of pre-marketing approaches, including *in vitro* and *in vivo* models, and the absence of validated biomarkers in clinical practice. The interest in idiosyncratic DILI is witnessed by the publication of the European Association for the Study of the Liver (EASL) guideline: a predictive algorithm assembling drug- and host-related factors, as well as mechanistic considerations, is awaited (European Association for the Study of the Liver, 2019). As expected in a safety field where randomized clinical trials are hardly feasible for ethical and methodological issues, the vast majority of recommendations stem from evidence graded to be of low quality (cases series and cohort studies), with the exception of immune checkpoint inhibitors, inducing hepatotoxicity in a substantial proportion of individuals, especially in combination regimens (homogeneous systematic reviews).

This review, not intended as comprehensive, aims at: 1) providing brief key insights into proposed mechanisms of idiosyncratic DILI, focusing on drug-related risk factors (physiochemical and pharmacokinetic properties); b) using cyclin-dependent kinase (CDK)4/6 inhibitors, relatively novel oral anticancer medications approved for breast cancer, as a case study to discuss the feasibility of early detection of DILI signals during drug development. Published data from pivotal clinical trials, unpublished post-marketing reports of liver adverse events, and pharmacokinetic properties will be used to provide a comparative evaluation of their liver safety and gain insight into drug-related risk factors likely to explain the observed differences.

## The Burden of Drug-Induced Hepatotoxicity: Clinical and Epidemiological Insights

The diagnosis of DILI is particularly challenging because it is largely based on exclusion of other causes: the timing of the onset of injury after the administration of implicated agent (latency), resolution after the drug is stopped (“dechallenge”), recurrence on re-exposure (rechallenge), knowledge of the agent’s potential for hepatotoxicity (likelihood), and clinical features (phenotype) are the major diagnostic elements (Hoofnagle and Björnsson, 2019). There are no diagnostic biomarkers routinely adopted in clinical practice, and special tests (liver biopsy, imaging, and testing for serologic markers) are helpful mostly in ruling out other causes of liver injury. Roussel Uclaf Causality Assessment Method (RUCAM) is a well-established score to assess causality in patients with suspected DILI (Danad and Teschke, 2018). This validated, transparent, and easily applicable tool has received worldwide appreciation, and important elements have been recently proposed to further increase its applicability and evaluate quality of planned studies (Teschke, 2019).

Determining the true incidence of hepatotoxicity by medicinal products remains problematic, also because epidemiological estimates depend on local pattern of prescriptions. Despite increasing awareness by stakeholders and advancements in designing less hepatotoxic compounds, the occurrence of hepatotoxic reactions appears to be stable over time, likely reflecting increased prescriptions and available medications (Garcia-Cortes et al., 2018).

The list of drugs associated with hepatotoxicity is ever-growing (Real et al., 2019) and includes not only drugs with widespread use in clinical practice, such as amoxicillin/clavulanate, anti-inflammatory agents, and statins, but also medications used in specialized settings, such as oncological agents and drugs for neurological diseases (Antonazzo et al., 2018; Raschi et al., 2019). However, a list based on the observed frequency of reporting is not indicative of the actual drug-related risk, especially because quality of reports in the published literature is unsatisfactory, and only a minority of reports is published in pharmacovigilance archives (Teschke, 2018). Bjornsson and Hoofnagle (2016) attempted to classify drugs listed in LiverTox® website based on the number of published case reports, an approach with uncertain reliability for novel drugs and, most importantly, unable to identify or propose medications with low DILI potential. A tentative “white list” informing on possible safe alternatives represent a current area of research. Therefore, the burden of DILI is likely. to be underestimated, also considering that: a) clinical trials are usually underpowered to identify rare idiosyncratic events, such as DILI; b) the majority of data are derived from post-marketing retrospective studies (e.g., spontaneous reporting system) (Raschi and De Ponti, 2015).

Different DILI studies are collecting prospective data through population-based design and dedicated registries: the former found large differences in the incidence rate (2.7 cases per 100,000 inhabitants in Delaware, 13.9 in France, and 19 in Iceland) and drug-related risks (highest for azathioprine and infliximab in Iceland); the latter consistently identified antibiotics as the most frequently implicated drug class and hepatocellular pattern as the most common pattern of liver damage (Alempijevic et al., 2017). Recent data from the Spanish and Latin American DILI network highlighted high prevalence of ibuprofen among causative drugs, with higher proportion of patients with diabetes, and severe DILI events (fatal/transplantation) (Zoubek et al., 2018). Notably, in mainland China, traditional Chinese medicines, herbal and dietary supplements, and antituberculosis medications emerged as the leading causes of DILI, with an estimated incidence in the general population of 23.8 per 100,000 inhabitants (retrospective design) (Shen et al., 2019).

The so-called herb-induced liver injury (HILI) is emerging as a worldwide epidemic, not only in Asia but also in the United States and Europe. Wong et al. (2019) reported an incidence as high as 81% in the Asia-Pacific region, whereas Wang et al. (2018b) found that, in China, traditional Chinese medicine accounted for almost 26% of DILI cases. This prompted the China Association of Chinese Medicine to publish guidelines for the evaluation of HILI (Wang et al., 2018a), as well as the creation of databases to organize the data on HILI aiming at both preventing future cases of liver injury from herbal medications and better comprehend their public health impact (Zhu et al., 2018; Liu et al., 2019). Hepatotoxicity of herbal and complementary medicines poses additional challenges in causality assessment, considering the lack of clinical specificity, frequent undeclared self-medication, and unclear composition of ingredients with potential contaminants. Red yeast rice and *Garcinia cambogia* have been recently identified as novel hepatotoxic compounds, possibly causing acute liver failure (Mazzanti et al., 2017; Crescioli et al., 2018), and an outbreak of acute noninfectious cholestatic hepatitis has occurred in Italy with turmeric-based dietary supplements (although an intrinsic hepatotoxicity is debated), thus further underlying the importance of pre-marketing quality assessment and post-marketing vigilance (Donelli et al., 2019).

## Is Liver Injury the Main Cause of Drug Attrition?

Drug development is facing a major paradigm shift, especially in oncology with the introduction of immunotherapy. The increasing demanding costs to achieve marketing authorization was not accompanied by a parallel increase in the rate of introduction of new molecular entities: the reported mean likelihood of approval for drugs entering clinical development for diverse disease areas during the period 2006 to 2015 was 9.6%, with a very low probability of success (<3%) for antineoplastic, immune-modulating, and nervous system agents (Waring et al., 2015). Although lack of efficacy continues to be the primary reason for phase III failure in recent years, unanticipated safety concerns in clinical trials represent a primary cause of failure (25%), particularly in phase II (Harrison, 2016).

DILI is erroneously considered to be among the most frequent causes of late-stage drug development interruption (Parasrampuria et al., 2018). The case of fasiglifam (TAK-875) is a paradigmatic example of the efforts required to genuinely identify drugs with clinically significant liver liability in drug development: the clinical program was halted only in late phase III after reviewing global clinical trial data (15 studies), documenting a combination of imbalance of alanine aminotransferase (ALT) elevations (mainly asymptomatic with two patients experiencing prolonged recovery) and three serious cases of liver injury (Marcinak et al., 2018). In addition, solithromycin, an antibiotic developed for treatment of community-acquired pneumonia, was not approved by the Food and Drug Administration (FDA) because many patients experienced transaminase elevations, even after 15 days of completing therapy (Buege et al., 2017), especially when the drug was administered intravenously. Intriguingly, solithromycin is now under investigation for its potential benefit in nonalcoholic steatohepatitis (NASH) because of its effect on the intestinal microbiome (Sumida and Yoneda, 2018).

Thus, the answer to the question in this section’s subtitle is probably no, and cardiotoxicity still remains one of the main reasons for drug development termination, both during pre-clinical and clinical stages. The reason for this most likely lies in the fact that significant advancement in understanding the mechanistic basis of cardiotoxicity prompted the refinement of new strategies with higher sensitivity and specificity to early detect cardiac liabilities (Gintant et al., 2016). The experience gained from drug-induced cardiotoxicity, especially pro-arrhythmia, can be applied in the next future to get the most from various ongoing DILI projects and set up a global response to efficiently optimize risk prediction (Raschi and De Ponti, 2017). Of note, a dedicated cardiovascular outcome trials on fasiglifam (demonstration of non-inferiority with respect to placebo on major adverse cardiovascular events is now mandatory for regulatory approval of novel antidiabetic drugs) enrolled 3,207 participants, but was prematurely stopped because of liver safety concerns, thus supporting the termination of relevant clinical program (Menon et al., 2018).

## Is Liver Injury the Main Cause of Drug Withdrawals After Marketing Authorization?

The imperfect prediction of DILI risk during drug development makes the post-marketing phase vital to early identification of liver safety signals (Raschi and De Ponti, 2015). Therefore, DILI represents a frequent reason for drug withdrawals in the post-marketing phase; a systematic review of 462 medicinal products withdrawn in the 1953 to 2013 period found that hepatotoxicity was the most frequent cause of drug withdrawal (18%), followed by immune-related reactions (17%), neurotoxicity (16%), and cardiotoxicity (14%) (Onakpoya et al., 2016). Notably, the supporting evidence consisted of anecdotal reports in 73% of cases.

On November 9, 2017, the Pharmacovigilance Risk Assessment Committee of the European Medicines Agency (EMA) recommended restrictions on the use of the daclizumab, with strict liver monitoring due to unpredictable and potential fatal immune-mediated liver injury up to 6 months after stopping the drug. On March 7, 2019, the EMA also recommended immediate suspension of the medicine’s marketing authorization in the EU and a recall of batches from pharmacies and hospitals following 12 cases of serious inflammatory brain disorders (three fatalities), including encephalitis and meningoencephalitis; the daclizumab case is paradigmatic of the importance of proactive post-approval safety monitoring to early and timely intercept idiosyncratic clinically significant cases of DILI (Antonazzo et al., 2018).

## Predictivity in Drug Development: From Pre-Clinical Assays to Clinical Trials

Animal models of DILI hold great promise and expectations for accurate prediction because they can theoretically gain insights into mechanistic bases. Besides ethical issues, the study of DILI in animals poses substantial technical challenges (pre-treatments potentially affecting the clinical relevance of the model, or genetic alteration designed to increase susceptibility to injury are required).

Three major approaches rely on induction of inflammation, suppression of immune tolerance, or genetic manipulation of mitochondrial function (McGill and Jaeschke, 2019). More recent approaches worked by impairing immune tolerance (the so-called Uetrecht-Pohl model) through depletion of myeloid-derived suppressor cells or inhibition of immune checkpoint receptors: the former approach was used in an animal model of halothane-induced liver damage, demonstrating a delayed onset of DILI accompanied by an infiltration of eosinophils in the liver (Chakraborty et al., 2015); the latter was achieved by blocking CTLA-4 or PD1 in the amodiaquine model first (Metushi et al., 2015), and subsequently demonstrated the ability to distinguish between hepatotoxic and non-hepatotoxic drugs of the same class (troglitazone vs pioglitazone, tolcapone vs entacapone) (Mak et al., 2018). However, there is no single animal model universally accepted to be highly predictive of DILI: inter-species differences in bile acid metabolism and reduced genetic variability have been identified as the main reasons for the high rate of false-negative results (Roth and Ganey, 2011; Ballet, 2015). Construct validity (i.e., how well the mechanism used to induce the disease phenotype in animals reflects the currently understood disease etiology and pathophysiology in humans) is the most problematic aspect to be faced by current research.

Overall, there are several *in vitro* models investigated in DILI research, especially for drug-induced cholestasis. However, their inherent limitations (lack of a complete immune system and cross-talk with other organs) make it unlikely that they will fully replace animals for DILI research. Several *in vitro* assay systems have been developed especially to investigate inhibition of hepatic transporters (see below): the reader is referred to an extensive review on the various pre-clinical models, differing in their goal, complexity, availability, applicability, and relevant predictivity to get closer to real hepatocyte phenotype (Petrov et al., 2018). Notably, it is difficult to foresee which model will optimally predict DILI risk, especially because our mechanistic understanding is oversimplified and conceptually flawed (Kenna and Uetrecht, 2018), thus making the use of multiple *in vitro* assays a strategy to be considered (Slizgi et al., 2016; Mosedale et al., 2017). Given that substantial evidence is converging toward an immune-related basis for DILI, future pre-clinical assays should determine immune response/tolerance to drugs to increase our prediction of DILI in drug development.

In clinical trials, predictivity of liver test monitoring is still limited, but elevated transaminases represent the first sign of injury. However, no adequate interval for monitoring is established as it depends on the evidence of hepatic risk (drug notoriety, e.g., monthly monitoring was found effective for isoniazid), and feasibility issues (European Association for the Study of the Liver, 2019).

The threshold for signal detection of DILI in clinical trials is still debated and evolving. In patients without underlying liver disease and liver chemistry being normal at baseline, DILI should be suspected if aminotransferases exceed 3× ULN, triggering close observation and workup for alternative causes. In 2011, a cutoff value of ALT > 5× ULN was proposed for DILI signal in the absence of liver-related symptoms or elevated serum total bilirubin (TBL) (Aithal et al., 2011). This higher threshold can reduce the likelihood of false positives: clinically insignificant and/or self-limited transaminases elevations/fluctuations in special populations (e.g., oncology), including patients with nonalcoholic fatty liver disease (NAFLD).

There is uncertainty related to identify and manage DILI signals in patients with pre-existing chronic liver disease, and debate exists whether these individuals have higher susceptibility to DILI (Chalasani and Regev, 2016). In this population, there is no consensus on how to determine the baseline transaminases levels, which should be measured at least twice before enrollment due to rapid fluctuation.

Use of multiples of baseline ALT rather than multiples of ULN was suggested as a threshold for suspecting DILI (Regev et al., 2019). Moreover, the relationship between DILI and NAFLD may be bidirectional (Bessone et al., 2018). Therefore, weekly monitoring should be considered in the early stage of development, focusing on signs/symptoms of “acute-on-chronic liver failure” and consulting hepatologists with expertise in DILI for causality assessment (Teschke and Danan, 2016).

Hy’s law still represents the most sensitive and specific predictor of a drug’s potential to cause severe hepatotoxicity, and is recognized as a key biomarker in drug development: the FDA’s Guidance on DILI, underlined that finding two Hy’s law cases during clinical development is highly predictive of severe DILI in the post-marketing phase (https://www.fda.gov/media/116737/download).

If liver tests exceeded thresholds, repeat testing should be done within 48 to 72 h, and the FDA guidance should be checked for discontinuation rules, which may have to be adapted, depending on the drug/disease under investigation and protocol.

However, ongoing efforts are exploring methods to improve prediction of serious hepatotoxicity using traditional tests. For instance, Robles-Diaz et al. (2014) proposed a new composite algorithm (AST > 17.3 × ULN, TBL > 6.6 × ULN, and AST/ALT > 1.5), which identified patients who developed acute liver failure with 82% specificity and 80% sensitivity. The latest international collaborative effort tested 14 candidate biomarkers and found that glutamate dehydrogenase appear to be more useful than microRNA-12 in identifying DILI patients, whereas total cytokeratin 18, osteopontin, and macrophage colony-stimulating factor receptor are promising prognostic candidates in acute DILI events (Church et al., 2019).

The use of multiple serum ALT measurements was investigated by the DILI-sim Initiative (Watkins, 2019), a public–private partnership that developed a proprietary mechanistic model (DILIsym), which predicts the time-dependent death of hepatocytes and relevant time-dependent concentration of serum biomarkers, typically ALT. The models were tested on cimaglermin alfa, a potential biological treatment for heart failure and suggested that the predominant mode of hepatocyte death was apoptosis rather than necrosis (ratio of caspase-cleaved K18 to FL-K18). The hepatocyte loss in two patients, estimated to be 6.6% to 12.4% (i.e., below the 30% observed in patients with severe DILI due to acetaminophen overdose), argued against considering these patients with bilirubin elevation actual Hy’s Law Cases (Longo et al., 2017). However, plotting the actual observed serum ALT versus time curves is not feasible in the real clinical practice. Therefore, a novel parameter was recently derived [P_ALT_ = ALT_AUC × Peak ALT^0.18^/10^5^ ((IU/L)^2^ × h)] to calculate the extent of hepatocyte loss during an acute DILI event through the maximum value and the AUC of serum ALT observed or estimated (Chung et al., 2019). By using data from patients with DILI (mimicking different scenarios of ALT changes), and assuming >10% hepatocyte loss as clinically significant, they proposed a P_ALT_ value of 15 as a critical point to identify patients with moderate-to-severe hepatic injury potentially representing actual Hy’s Law cases. In the clinical setting, even in a trial, a complete time course of ALT measurements may be unavailable, and recovery is incomplete. Therefore, both best- and worst-case scenarios were hypothesized, depending on whether or not the underlying drug-induced necrosis is assumed to continue. This promising approach, if confirmed, may be used to early prevent severe DILI event before actual occurrence; in other words, it is not necessary to actually observe a Hy’s law case to indicate potential of a new drug to trigger liver failure.

## Etiology of DILI: the Role of Drug Properties

Although different hypotheses have been put forward to explain the complex and multifactorial basis of idiosyncratic DILI, our mechanistic understanding is partial and oversimplified. A detailed description of the various hypotheses was already extensively covered and beyond the aim of this review. Notably, the various theories are not mutually exclusive (sometimes are even complementary), and converge on the involvement of adaptive immune system *via* different interconnected pathways (Mak and Uetrecht, 2017; Mosedale and Watkins, 2017; Roth et al., 2017). Moreover, additional non-immune hypotheses have been postulated, including mitochondrial injury and bile salt export pump (BSEP) inhibition.

The involvement of the immune system is corroborated by different lines of evidence: 1) the large number of genome-wide association studies and candidate gene approaches identifying specific genetic susceptibility and correlations between human leukocyte antigen (HLA) polymorphisms and DILI occurrence (e.g., for flucloxacillin, amoxicillin/clavulanate, minocycline, ximelagatran, lumiracoxib (Kaliyaperumal et al., 2018); 2) the delayed onset of DILI, typically after 1 to 6 months of continuous treatment, worsening or even initiating after drug interruption; 3) the prompt recurrence of DILI upon drug rechallenge with the offending drugs, after complete recovery; 4) frequent manifestation of DILI as drug-induced autoimmune hepatitis sharing many clinical features with idiopathic autoimmune hepatitis (e.g., anti-TNF agents). In this challenging scenario, the differential diagnosis can be resolved *a posteriori* once remission with corticosteroids is achieved: drug-induced autoimmune hepatitis does not usually relapse after withdrawal of immunosuppressive therapy.

**Figure 1:**
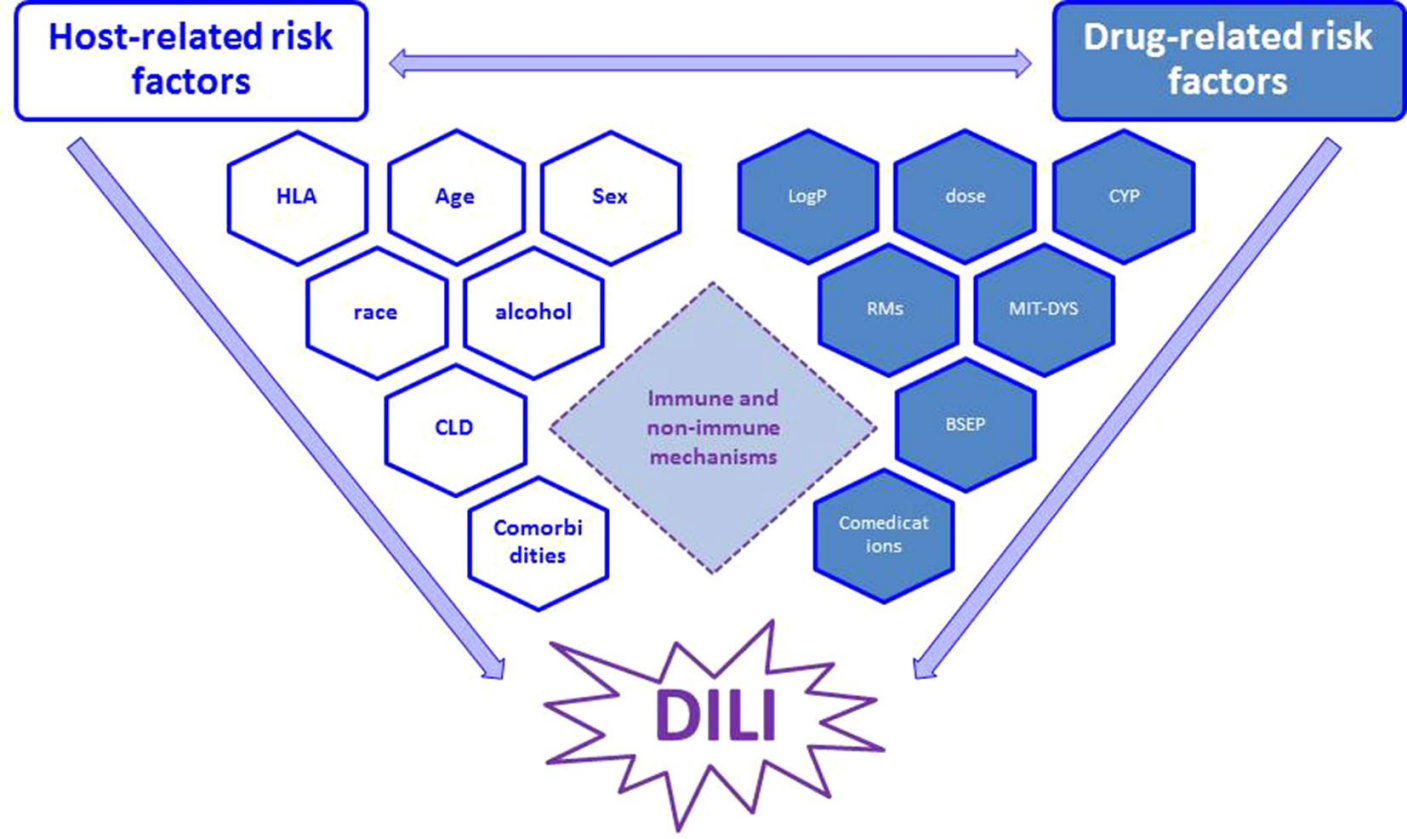
Interplay between main host- and drug-related risk factors, which may synergize and finally result in occurrence of DILI through immune and non-immune mechanisms (see text for details). RM, reactive metabolites; CYP, cytochrome (indicating metabolic pathway); MIT-DYS, mitochondrial dysfunction; BSEP, bile salt export pump.

The common view postulated that an interplay between drug properties and host factors may play a crucial role in occurrence of DILI (Fontana, 2014; Chen et al., 2015): susceptibility to hepatotoxicity may be increased by specific drug’s pharmacological action and/or pharmacogenomics (**Figure 1**) (Kaliyaperumal et al., 2018). This may explain why trovafloxacin or nimesulide are hepatotoxic (as compared to levofloxacin or other nonsteroidal anti-inflammatory drugs, respectively) (Roth and Ganey, 2010). While there is no consensus on the real contribution of host-related risk factors (age, sex, race, alcohol, pregnancy, comorbidities) on DILI occurrence (although some of them are now included in the RUCAM causality assessment), the role of pharmacological risk factors is recognized, especially in drug development. Among drug properties, recognized factors contributing to initial cell damage include: threshold dose, lipophilicity, formation of reactive metabolites (RMs), oxidative stress, mitochondrial liability, hepatic drug metabolism, and inhibition of hepatic transporters (Begriche et al., 2011; Chen et al., 2015), which are also recommended by the latest EASL 2019 guidelines on DILI (evidence: Extrapolation from 2c studies (outcomes research and mechanistic studies)” (European Association for the Study of the Liver, 2019).

There is a large body of evidence indicating that RMs are implicated in the pathogenesis of DILI and other idiosyncratic adverse events (Cho and Uetrecht, 2017). Therefore, during drug development, particular attention is paid to structural alerts (SAs), which may be responsible for both idiosyncratic toxicities and pharmacological action of certain drugs (Stepan et al., 2011; Limban et al., 2018). In particular, aromatic rings are considered major culprits for RM formation of drugs with DILI risk in humans (Claesson and Minidis, 2018). Different Web-based platforms have been proposed for collecting and storing toxicological SAs publicly available from literature and for virtual screening of chemical libraries to flag potentially toxic chemicals and compounds (Sushko et al., 2012). Unfortunately, RMs alone cannot explain the whole picture: there are drugs, such as ethacrynic acid, that are chemically reactive but virtually never cause DILI, whereas agents, such as allopurinol and pyrazinamide, can elicit idiosyncratic reactions but do not appear to form RMs. Moreover, there is no method to determine which RM is responsible for a given idiosyncratic reactions, and, most importantly, the relationship between RM formation and actual DILI occurrence is neither straightforward nor precise (Cho and Uetrecht, 2017).

The fact that the dose (for drugs administered orally) may play a role in DILI was highlighted in 1999 by Uetrecht (1999), later demonstrated using 598 DILI cases reported in Sweden, where an oral daily dose ≥50 mg was noted in 77% of the cases (Lammert et al., 2008) and further corroborated by Spanish and Icelandic data (Lucena et al., 2009; Bjornsson et al., 2013). Notably, the threshold dose may have substantial inter-individual variability (DILI may occur when increasing the dose, still within the recommended daily dose range) (Carrascosa et al., 2015), and DILI induced by agents used at daily dose higher than 50 mg was found to have significantly shorter latency period as compared with DILI caused by drugs taken at lower dosage (38 vs 56 days) (Vuppalanchi et al., 2014). Antibiotics are an exception: amoxicillin-clavulanate (daily dose >1000 mg) was associated with a particular phenotype of “delayed onset of DILI,” a term referring to the delay in DILI manifestations after interruption of the agent, to be distinguished from long latency time during drug administration (Gonzalez-Jimenez et al., 2019). Therefore, the question arises as to whether or not high dose is just an epiphenomenon, and DILI simply overlaps with most frequently prescribed medications used at recommended doses.

Different studies have investigated the relationship between daily dose and lipophilicity (often measured as the log of octanol–water partition coefficient [LogP]), referred to as the “rule-of-two” (RO2): drugs with highly lipophilicity (logP ≥ 3) and a daily dose ≥100 mg/d are associated with increased risk of DILI (Chen et al., 2013). Notably, lipophilicity also correlates with drug attrition: compounds failing owing to clinical safety in phase I are significantly more lipophilic compared to those successfully progressing to phase II (Waring et al., 2015). The RO2 has been therefore proposed as a simple predictive tool to discriminate hepatotoxic drugs during drug development, as demonstrated for direct-acting antivirals used in chronic hepatitis C (Mishra and Chen, 2017), although lipophilicity was not confirmed as independent DILI risk factor in a study on 975 oral drugs (Weng et al., 2015). It was hypothesized that higher lipophilicity could facilitate metabolism by hepatocytes resulting in increased amounts of RMs (Chen et al., 2015), thus making LogP a surrogate for liver exposure to RMs. In fact, both lipophilicity and drug metabolism (extensive liver metabolism, for drugs that are P450 substrates or inhibitors) were found to be independent DILI risk factors, especially in conjunction with dose (Yu et al., 2014; McEuen et al., 2017). In an effort to create an algorithm unifying the aforementioned drug properties, Chen et al. (2016a) developed a score model to predict the severity of DILI in humans, basically by factoring RM into the RO2 and using data on the dose and Cmax. High molecular weight (>600 Da) and low polarity (topographical Polar Surface Area (<75 Å2) were also demonstrated to be additional physiochemical properties correlating with the likelihood of having DILI in *in vivo* models (Hughes et al., 2008; Greene et al., 2010; Yucha et al., 2017).

Mitochondrial dysfunction was proposed as a major pathway whereby drugs and/or their metabolites can induce liver injury. Different mechanisms of mitochondrial impairment have been described so far, including mitochondrial permeability transition pore (MPTP) opening and direct impairment of oxidative phosphorylation process, which can induce necrosis and/or apoptosis thereby leading to cytolytic hepatitis, as well as inhibition of mitochondrial fatty acid oxidation (FAO), by direct inhibiting FAO enzyme, indirectly compromising mitochondrial respiratory chain activity, and causing depletion or damage of mitochondrial DNA, thus finally resulting in microvesicular steatosis and steatohepatitis (Begriche et al., 2011; Ramachandran et al., 2018). Notably, several drugs, including amiodarone, diclofenac, tamoxifen, valproic acid, and zidovudine, act *via* multiple mechanisms. A comprehensive analysis of 228 compounds found that the predictive performance of the mitochondrial assays is superior for hepatotoxicity versus cardiotoxicity and nephrotoxicity, when the analysis was done at 100* Cmax, and LogP emerged to be significantly associated with mitochondrial toxicity (Rana et al., 2019). However, the hypothesis that mitochondrial injury plays a role in DILI was challenged as there are drugs that impair electron transport chain without causing DILI (Cho et al., 2019). Inhibition of the BSEP by drugs or their metabolites has been identified as a major risk factor for *in vivo* DILI prediction and is thought to be an important mechanism leading to drug-induced cholestasis. For a wide variety of drugs (cyclosporine A, rifampicin, bosentan, troglitazone), a correlation was observed between the potency of *in vitro* BSEP inhibition and its propensity to cause DILI in humans, although discrepancies exist in which methods to use and the extent to which BSEP inhibition predicts clinical DILI (Yucha et al., 2017); moreover, it must be underlined that correlation does not prove causation. BSEP inhibition *per se* is not indicative of the actual DILI risk and thus cannot be used as a standalone evidence for stopping drug development. When interpreting *in vitro* BSEP inhibition data, it is critical to consider both potency of inhibition (as expressed by the Ki value or with limited information by the IC_50_ value) and *in vivo* drug exposure. Both parameters are challenging to be determined experimentally, and there is debate on the BSEP IC_50_ cutoff values to identify “concerning level” of BSEP inhibition (from 25 to 300 µM). The latest international transporter consortium perspective proposed IC_50_ < 25 µM during the drug discovery phase to indicate the need to further explore potential correlation with maximum total plasma concentration (C_ss, plasma_) during early clinical drug development (Kenna et al., 2018). For instance, a common observation was that drugs exhibiting C_ss, plasma_/BSEP IC_50_ > 0.1 and administered systemically for prolonged use caused DILI in humans. Interestingly, compounds that are dual inhibitors of BSEP and mitochondrial function were found to potentially correlate with acute liver failure (Aleo et al., 2014). It was also observed that the great majority of compounds that have been associated with DILI and are BSEP inhibitors are also Biopharmaceutics Drug Disposition Classification System class 2 drugs (highly metabolized and poorly soluble) (Chan and Benet, 2018). When BSEP function is impaired, basolateral efflux systems through multidrug resistance proteins (MRP2, MRP3, and MRP4) are theoretical salvage systems to lower the burden of bile salts and drug metabolites for hepatocytes. However, their role as additional susceptibility factors for DILI is debated because of conflicting findings (Yucha et al., 2017).

The role of concomitant drugs warrants a final remark. In clinical practice, determination of the most likely causative agent is an achievable clinical task (based on the known hepatotoxic potential and temporal consideration), although disentangling the role of drug interactions in DILI onset among poly-treated individuals is not straightforward, apart from the case of a drug with recognized modulating effect on metabolism (inhibition or induction of cytochrome activity). The analysis of the WHO pharmacovigilance database described the liver event reporting frequency of drugs commonly associated with hepatotoxicity and suggested both beneficial (TNF inhibitors/folic acid and isoniazid) and detrimental (e.g., proton pump inhibitors and amoxicillin/clavulanate) interactions (Suzuki et al., 2015). However, it remains uncertain whether the potential hepatic modulation is related to co-medications or the underlying disease requiring concomitant drugs.

## Pharmacology At Work for DILI Prediction: a Case Study on CDK4/6 Inhibitors

CDKs were expected to be key therapeutic targets in cancer and pan-CDK inhibitors were initially designed, albeit with disappointing results (Asghar et al., 2015). The development of selective of CDK4/6 inhibitors has markedly changed the perception of CDKs as therapeutic targets in cancer and, based on favorable data from pivotal phase III trials, palbociclib, ribociclib, and abemaciclib, are now approved both in the United States and Europe for women with hormone receptor-positive, human epidermal growth factor receptor 2-negative advanced breast cancer. While all agents significantly increased progression free survival (absolute median gain about 15 months) when added to endocrine therapy in first- and second-line settings, palbociclib, the first-in-class medication, resulted in longer overall survival only in patients who had sensitivity to previous endocrine therapy (median, 39.6 months; with a gain of 10 months) (Turner et al., 2018), whereas ribociclib plus endocrine therapy resulted in significantly longer overall survival than endocrine therapy alone (estimated overall survival at 42 months was 70.2%), even in subjects receiving an aromatase inhibitor (Im et al., 2019). Although these data are promising, it is still premature to conclude about actual different efficacy, especially because post-marketing effectiveness is currently underexplored.

In general, CDK4/6 inhibitors share different chemical and pharmacokinetic features, including metabolism mediated by CYP3A4 (with production of intermediate active metabolites, potentially resulting in drug-drug interactions), and biliary clearance as the main elimination pathway (**Table 1**). From a safety standpoint, they are well-tolerated agents, with similar safety profile, although some differences exist in the pattern and frequency of toxicities, which might influence the choice of a given medication.

**Table 1:** Comparative analysis of key chemical properties and pharmacokinetics of CDK4/6 inhibitors. Data were retrieved from following references: Curigliano et al., 2017; De et al., 2018; Robert et al., 2019; Roskoski, 2019.

	Ribociclib	Abemaciclib	Palbociclib
***Chemical properties***			
**Molecular weight (Da)**	434,548	506,606	447,543
**Polar surface area**	91.2	75	103.35
**Ring count**	5	5	5
**Primary pharmacophore structure**	2-amino-pyrrolo[2,3-d]pyrimidine	amino-pyrimidine-benzimidazole	amino-pyrido[2,3-d]pyrimidine
**Major secondary drug components**	diaryl-amino group	diaryl-amino group	diaryl-amino group
***Pharmacokinetic parameters***			
**Recommended daily dose (mg)**	600	300	125-200
**Schedule**	3 weeks ON/1 week OFF	Continuous	3 weeks ON/1 week OFF for 125 mg (2 weeks ON/1 week OFF for 200 mg)
**C_max_ (ng/mL)**	2100 (assuming the worst case scenario of accumulation after 21 days of treatment during scheduled treatment cycle)	249 (mean value at steady state after 300 mg daily)	194 (mean value at day 8, after 150 mg daily)
**T_max_ (h)**	1–5	5–6	4.2–5.5
**V_d_ (l)**	1090	690.3	2793
**t_1/2_**	32.6	17-38	25.9
**Metabolism**	CYP3A4	CYP3A4	CYP3A and SULT2A1
**Active metabolites**	Yes (LEQ803, CCI284)	Yes	No
**Elimination**	Biliary (negligible renal excretion)	Biliary (negligible renal excretion)	Biliary (negligible renal excretion)

The most common side effect for palbociclib and ribociclib is neutropenia, whereas gastrointestinal toxicity is associated especially with abemaciclib (showing less selectivity for CDK4, which plays a critical role in hematopoietic stem cell differentiation). These differences may be particularly relevant in the adjuvant setting where even grade 2 diarrhea may be considered unacceptable compared with asymptomatic grade 4 hematological toxicities (Cho and Lee, 2018). Higher frequency of QT prolongation emerged for ribociclib, whereas increased liver enzymes was recorded with ribociclib and abemaciclib resulting in regulatory warnings, with specific recommendations for monitoring liver function test (to be checked before and during treatment), eventual dose interruption or drug discontinuation (Thill and Schmidt, 2018).

These recommendations on liver monitoring under ribociclib originated from data from pivotal phase III trials, in particular MONALEESA-2, where four cases of Hy’s law were confirmed (three suspected to be related to study treatment) (Hortobagyi et al., 2016): two cases showed findings on biopsy suggestive of autoimmune hepatitis, without fatalities or hepatic failure with permanent disability. Liver function tests for all four patients recovered to normal levels 98 to 154 days following drug discontinuation. **Table 2** provides an overview of data on liver safety from phase III trials of CDK4/6 inhibitors; this comparative analysis underlines that, although liver injuries meeting criteria for Hy’s law definition have been described only for ribociclib, cases of liver injury (increased ALT and/or AST levels, even serious) have been reported in significantly higher proportion as compared with control arms, especially for abemaciclib. Accordingly, the European summary of product characteristics and US product information contain warnings on hepatobiliary toxicity for both agents.

**Table 2:** Comparative incidence of liver injuries with CDK4/6 inhibitors from pivotal phase III trials. In parentheses, percentages are indicated. Data were retrieved from the following references: Turner et al., 2015; Cristofanilli et al., 2016; Finn et al., 2016; Hortobagyi et al., 2016; Goetz et al., 2017; Sledge et al., 2017; Hortobagyi et al., 2018; Slamon et al., 2018; Tripathy et al., 2018; Turner et al., 2018; Im et al., 2019.

	Ribociclib			Abemaciclib		Palbociclib	
	MONALEESA-2	MONALEESA-3	MONALEESA-7 €	MONARCH-2	MONARCH-3	PALOMA-2	PALOMA-3
**Population**	HR+/HER2−, post-menopausal women with advanced breast cancer	HR+/HER2−, with advanced breast cancer who were treatment naïve or had received up to one line of prior endocrine therapy in the advanced setting	HR+/HER2−, premenopausal women with advanced breast cancer	HR+/HER2−, with advanced breast cancer who had progressed while receiving neoadjuvant or adjuvant endocrine therapy	HR+/HER2− postmenopausal women with advanced breast cancer who had no prior systemic therapy in the advanced setting	HR+/HER2− post-menopausal women with advanced breast cancer previously untreated	HR+/HER2− women with advanced breast cancer that had relapsed or progressed during prior endocrine therapy
**Intervention/comparator**	Ribociclib plus letrozole/letrozole	Ribociclib plus fulvestrant/placebo plus fulvestrant	Ribociclib plus endocrine therapy/placebo plus endocrine therapy	Abemaciclib plus fulvestrant/fulvestrant	Abemaciclib plus a nonsteroidal aromatase inhibitor/placebo plus a nonsteroidal aromatase inhibitor	Palbociclib plus letrozole/placebo plus letrozole	Palbociclib plus fulvestrant/placebo plus fulvestrant
**Increased ALT any grade (%)**	52 (15.6) vs 13 (3.9)^‡^	No data	43 (13) vs 25 (7)	59 (13.4) vs 12 (5.4)	149 (47.6) vs 39 (25.2)	No cases reported†	19 (6) vs 6 (3)
**Increased ALT grade 3/4 (%)**	31 (9.3) vs 4 (1.2)	41 (8.5) vs 1 (0.4)	18 (5) vs 5 (1)	18 (4.1) vs 4 (1.8)	22 (7.0) vs 6 (1.9)	1 had grade 4 event	6 (2) vs 0 (0)
**Increased AST any grade (%)**	50 (15.0) vs 12 (3.6)	No data	42 (12) vs 30 (9)	54 (12.2) vs 15 (6.7)	115 (36.7) vs 36 (23.2)	No cases reported	40 (11.6) vs 13 (7.6)^$^ 24 (7) vs 8 (5)
**Increased AST grade 3/4 (%)**	19 (5.7) vs 4 (1.2)	29 (6.0) vs 2 (0.8)	12 (4) vs 4 (1)	10 (2.3) vs 6 (2.7)	12 (3.8) vs 1 (0.6)	No cases reported	11 (3.2) vs 4 (2.3)^$^ 9 (3) vs 3 (2)
**Hy’s Law**	Four cases (three suspect to be drug-related)*	Two cases^#^	No cases reported	No cases reported	No cases reported	No cases reported	2 patients with hepatic failure

^€^in the overall survival analysis, grade 3/4 “hepatobiliary toxicity” was documented in 11% vs 6.8%, whereas all grade “hepatobiliary toxicity” was recorded in 27.5% vs. 22.8% (Im et al., 2019).

^‡^in the updated results from MONALEESA-2 the following data on abnormal Liver Function Tests (increased alanine aminotransferase, increased aspartate aminotransferase, increased blood bilirubin) emerged: any grade: 67 (20.1%) vs. 21 (6.4%); grade 3/4: 34 (10.2%) vs 8 (2.4%).

^†^regardless of severity, three patients with ALT elevation required dose reduction/interruption in the palbociclib group.

^$^based on the overall survival analysis (Turner et al., 2018).

* two cases with features of autoimmune hepatitis; none of these cases resulted in death, and aminotransferase and bilirubin levels returned to normal in all four patients within 98-154 days after the discontinuation of ribociclib. Two of these four cases showed findings on biopsy suggestive of autoimmune hepatitis.

^# ^liver enzymes normalized after discontinuation.

Based on these data from pre-marketing clinical trials, there is interest in determining the existence of a specific drug-related liver toxicity rather than a class effect. Therefore, we carried out a two-fold “from bedside to bench” approach.

First, we used the public dashboard of the FDA adverse reporting system (FAERS) to extract adverse events suspectedly attributed to CDK4/6 inhibitors and describe their current reporting pattern in the real-world (https://www.fda.gov/drugs/questions-and-answers-fdas-adverse-event-reporting-system-faers/fda-adverse-event-reporting-system-faers-public-dashboard, searches performed on 07/06/2019; data as of March 31, 2019). It is important to quickly remind here about the drawbacks of this analysis, especially considering the different time on the market of the three anticancer drugs. Because of these inherent limitations, including data quality (potential existence of pre-marketing reports, duplicates, and missing information), the likelihood of under-reporting, the potential influence of external factors (time on the market and media attention), the lack of exposure data (drug prescription/consumption), and inability to establish firm causality, incidence, risk assessment, and risk ranking cannot be provided. These data only provide a general picture of the current liver reporting pattern with CDK4/6 inhibitors. Second, we collected publicly available information on chemical and pharmacokinetic features of CDK4/6 inhibitors, previously discussed to likely contribute to DILI occurrence: this critical insight into pharmacovigilance and pharmacological data would potentially reveal differences among the three compounds and related contributing factors to advise on further investigation for better understanding the mechanistic basis.

The ancillary analysis of the FAERS database (**Table 3**) indicates that: (a) the global post-marketing reporting pattern is in line with safety profile observed in pre-marketing clinical trials, with gastrointestinal and hematological effects being the most common toxicities of CDK4/6 inhibitors; (b) the frequency of liver injuries (percentage as compared to total reports) for ribociclib is higher as compared with the relevant proportions for abemaciclib and especially palbociclib, although the proportion of serious liver reports (i.e., those resulting in death, hospitalization—initial or prolonged—being life-threatening or leading to disability/congenital anomaly) is comparable among CDK4/6 inhibitors (range, 62–70%). Please note that a notoriety bias (i.e., increased reporting of liver injuries due to media attention or increased awareness of submitters) cannot be ruled out. Notably, the proportion of hepatic failure was substantially higher for palbociclib, whereas four cases of autoimmune hepatitis were recorded for ribociclib. These findings, in agreement with DILI signal emerging from pivotal trials, strengthened the importance of verifying the immune-mediated hypothesis.

**Table 3:** Summary of spontaneous reports recorded in the FDA Adverse Event Reporting System (FAERS). See text details.

	Ribociclib	Abemaciclib	Palbociclib
***Total reports***	2551	1114	27,771
**Age distribution**			
**18–64 years**	600	237	9945
**65–85 years**	525	255	11,041
**>85 years**	24	24	662
**Not specified**	1390	598	6102
**Serious cases**	1785	434	13,416
**Death**	296	68	2880
**Most frequent adverse event**	Neutropenia (n = 252; 9.8%)	Diarrhea (n = 408; 36.6%)	Fatigue (n = 5168; 18.6%)^#^
***Liver reports*^‡^**	*197 (7.7%)*	*29 (2.6%)*	*320 (1.2%)*
**Serious liver reports ^†^**	143 (72.6%)	18 (62.1%)	224 (70.0%)
**ALT increase/abnormal^†^**	65 (33%)	4 (14%)	118 (37%)
**AST increase/abnormal^†^**	58 (29%)	5 (17%)	125 (39%)
**γ-GT increase/abnormal^†^**	22 (11%)	0	33 (10%)
**AP increase/abnormal^†^**	11 (5.6%)	3 (10%)	43 (13.4%)
**Hepatic failure^†^**	11 (5.6%)	2 (6.9%)	83 (25.9%) ±
**Autoimmune hepatitis ^†^**	4 (2%)	0	1

^#^ Neutropenia (N=2235; 8%).

^‡^ Including AST/ALT elevation, liver function abnormalities, transaminases increased, bilirubin elevation; percentage out of total reports. Please note that a single liver report may contain more than one liver event.

^†^ Percentage out of liver reports. Seriousness defined as causing death, being life-threatening, requiring hospitalization (initial or prolonged), or leading to disability or congenital anomaly.

^±^ the following hepatic events were reported: acute hepatic failure (n = 17), pseudocirrhosis (n = 12).

A synopsis of collected physiochemical and pharmacological features is presented in **Table 4**. From a chemical viewpoint, public online prediction tools, namely, ADVERPred (http://www.way2drug.com/adverpred/, also providing a probability that a drug is “active” in terms of DILI, arrhythmia, myocardial infarction, cardiac failure, and nephrotoxicity) and HepatoPred (http://ccsipb.lnu.edu.cn/toxicity/HepatoPred-EL/index.html, specifically developed to test the hepatotoxicity liability) were used to verify whether CDK4/6 inhibitors share similar “pharmacophores,” suggesting DILI risk: they were all classified as “potential hepatotoxic”, with the exception of abemaciclib resulting as “uncertain” in ADVERPred. These findings suggest that the three agents are likely to possess SAs and generate RMs, which have recognized role in idiosyncratic DILI occurrence, although there are no dedicated published studies to our knowledge.

**Table 4:** Pharmacological properties of CDK4/6 inhibitors that may play a role in DILI occurrence. See text for details.

Features	Ribociclib	Abemaciclib	Palbociclib
***Physiochemical factors***			
**Molecular weight (> 600 Da)**	X (435)	X (506)	X (447)
**Max daily dose (≥50–100 mg/die)**	√ (600)	√ (300)	√ (125)
**Lipophilicity (LogP≥3)**	X (2.62)	√ (4.25)	X (2.12)
**Topological polar surface area (< 75 Å2)**	X (91.21)	X (75)	X (103.35)
**C_plasma_ [max tot conc]/BSEPIC_50_≥0.1]**	√ (0.4468)	Cannot be calculated	X
***Oxidative stress***			
**Reactive metabolite formation**	There are no public data		
**ROS induction/GSH depletion α**			
Mitochondrial liability*			
**Mitochondrial dysfunction**	There are no public data		Negative mitochondrial liability^‡^
***Metabolism and hepatic transporters***			
**Hepatic metabolism**	√	√	√
**BSEP inhibition (IC_50_ < 25-300 µM)**	√ [4.7 (3.9 for metabolite CII284)	No data	X (does not inhibit BSEP)
**Dual BSEP and other efflux transporters inhibition (MRP2, MRP3, or MRP4)**	√	No data	No data
**MRP2 (IC_50_µM)**	> 300 (> 50 for intermediate metabolites) [EPAR]	No data	No data
**MRP3 or MRP4**	No data	No data	No data
**MATE1 (IC_50_µM)**	1.7 (*in vivo* relevance); 0.3–0.6 for intermediate metabolites [EPAR]	Potential relevance but no specific data are provided [EPAR]	No data
***DILI risk scores and prediction***			
**ADVERPred**	0.647 (active)	0.422 (uncertain)	0.535 (active)
**HepatoPred**	0.66 (hepatotoxic)	0.67 (hepatotoxic)	0.63 (hepatotoxic)
**Dose-based DILI risk score**	4.48–7.32^#^	4.43–7.27^#^	3.70–6.54^#^
**Cmax-based DILI risk score**	3.48–6.29^#^	2.90–5.71^#^	2.14–4.95^#^

√ = CRITERIUM FULFILLED; X = CRITERIUM NOT FULFILLED. In parentheses, specific data are provided.

* Based on the following abilities/features: protein binding (expoxidation), MPTP (mitochondrial permeability transition pores) opening, direct inhibition of mitochondrial FAO (fatty acid oxidation), OXPHOS (oxidative phosphorylation) uncoupling, direct inhibition of mitochondrial respiratory chain (MRC), mitochondrial DNA (mtDNA) depletion/damage.

^‡^ Based on in vitro assays performed for 288 drugs and testing up to 100*Cmax (Rana et al., 2019).

^#^ Calculated based on formulas by Chen et al. (2016a). The highest score represents the worst case scenario, assuming formation of reactive metabolites.

EPAR: European public assessment report.

However, the RO2 failed for ribociclib and palabociclib, but was positive for abemaciclib, likely due to the high lipophilicity of the drug. In the light of these discrepancies and considering that the DILI risk model by Chen et al. (2016a) is particularly useful for agents with similar chemical structure and pharmacodynamics but divergent toxicities, we applied this risk model using collected data on dose and Cmax: the three CDK4/6 inhibitors received comparable scores, the highest being 7.32 for ribociclib (dose-based score), the lowest being 4.95 for palbociclib (Cmax-based score), assuming RM formation. According to the original study by Chen et al. (2016a) drugs receiving a score of 6 are hepatotoxic in humans, whereas drug with less DILI concern/weak evidence generated a score of 4. Therefore, CDK4/6 inhibitors are predicted to cause hepatotoxicity in humans.

As regard drug-related properties potentially indicating non-immunological DILI mechanisms, only palbociclib was tested for mitochondrial toxicity and emerged with negative liability in *in vitro* assays up to 100*Cmax (Rana et al., 2019). Conversely, ribociclib is a recognized inhibitor of hepatic transporters (*in vitro* inhibition with *in vivo* relevance), especially BSEP. In particular, ribociclib causes dual inhibition of BSEP and basolateral efflux systems (e.g., MRP2 and MATE1), which are additional susceptibility factors for cholestasis. Conversely, palbociclib does not inhibit BSEP, and the inhibitory effect of abemaciclib on these transporters was not tested during drug development, and no data are published so far to our knowledge.

## Conclusion and Perspectives

Idiosyncratic DILI is a current challenge for drug developers, as demonstrated by the various collaborative initiatives (public–private partnerships) aiming at minimizing drug attrition. The quest for predictive tools to *a priori* identification of both host factors and pharmaceutical features conferring a DILI risk has led to the development of numerous cell-based systems, animal models, and *in silico* algorithms. Notwithstanding these efforts, our mechanistic understanding is still imperfect, and both immune and non-immune factors may synergistically interact by increasing DILI susceptibility and its eventual occurrence.

In the recent past, different pharmacological properties have been proposed as potential risk factors for DILI, with lipophilicity, formation of RMs, and BSEP inhibition emerging as recognized features contributing to initial cell damage: their accurate pre-marketing appraisal is recommended (European Association for the Study of the Liver, 2019). Considering the limitations of pre-clinical assays (*in vitro* findings do not inform on the underlying mechanism, and correlation does not necessarily mean causation), these data should not be viewed as a “stop-or-go” criterion toward marketing authorization, but rather as an early risk minimization strategy to direct post-marketing phase and timely intercept a potential liver signal. At present, there is room for improvement both in the FDA and EMA regulatory guidelines; the former, published in July 2009, addresses only the pre-marketing clinical evaluation (https://www.fda.gov/media/116737/download), whereas the latter, dated July 2010, is a reflection paper providing a general perspective \and stating that “standard non-clinical toxicity studies are the cornerstone of preventing of hepatotoxicity in humans” (https://www.ema.europa.eu/en/non-clinical-evaluation-drug-induced-liver-injury-dili). Conversely, both FDA (https://www.fda.gov/media/82734/download) and EMA (https://www.ema.europa.eu/en/documents/scientific-guideline/guideline-investigation-drug-interactions_en.pdf) guidelines on the pre-marketing investigation of drug–drug interactions specifically recommend testing drugs for BSEP inhibition and other hepato-biliary transporters. An updated unified comprehensive DILI guideline is warranted to create an integrated DILI risk assessment (Kenna et al., 2018).

In the current era of artificial intelligence, a global collaborative response from existing consortia is desirable: data sharing *via* public repositories will increase our mechanistic understanding, enhance early prediction, and allow timely recognition during drug development, thus finally achieving successful DILI prevention and assessment in the pre-marketing phase. In parallel, a comprehensive DILI classification scheme is needed to have a common centralized repository such as the one assembled by the Liver Toxicity Knowledge Base (LTKB) project, which has created the largest reference drug list ranking (the so-called DILIrank) by annotating severity and causality of 1036 drugs (Chen et al., 2016b). The LTKB is now working to merge various data sources (e.g., LiverTox) to have a common data set for developing biomarkers and predictive models through emerging technologies (Thakkar et al., 2018).

From a clinical viewpoint, algorithms for assessing hepatocyte loss, detecting subclinical episodes of liver damage, predicting, and preventing severe liver toxicity have been proposed or are being developed, but warrant further validation and testing in a large sample of patients and data sets (Kullak-Ublick et al., 2017). The choice of biomarkers is also a major challenge taken up by numerous initiatives worldwide. Among these, the International Network of Drug-induced Liver Injury Research, led by Alexander Gerbes (LMU Munich), is evaluating the performance of the monocyte-derived hepatocytes generated from patients with DILI (MH cell test) in different European and Asian cohorts (https://www.game-med.net/dili), with promising results, as compared with RUCAM, in discriminating the actual culprit agent in subjects taking multiple medications (Benesic et al., 2016; Benesic et al., 2018).

The case of CDK4/6 inhibitors can be used as a paradigm for critical assessment of pharmacological properties before approval: the use of easily applied DILI risk scores predicted a hepatotoxic risk in humans and should be viewed as a risk minimization strategy both for drug development and post-marketing monitoring. The inhibitory effects on hepatic transporters, namely BSEP, represent the key pharmacological feature of ribociclib, especially as compared with palbociclib, which might explain, at least in part, the higher frequency of liver reports both in pivotal clinical trials and in the post-marketing phase. Although there are no published data on transporter inhibitions by abemaciclib, its high lipophilicity might potentially account for the observed frequency of liver events (clinical trials and post-marketing reports), comparable to that observed for ribociclib. While inhibitory property on hepatic transporters by ribociclib potentially suggests the hypothesis of a non-immunological mechanism, cases of autoimmune hepatitis emerging from MONALEESA-2 trial and recorded in the FAERS database do not allow to rule out an immune-related etiology and strengthen the importance of: a) investigating the role of immune tolerance using the aforementioned animal models; b) conducting multicenter post-authorization safety studies specifically devoted to assess liver safety in subjects using CDK4/6 inhibitors. In fact, despite possessing the most favorable pharmacological property (lack of mitochondrial liability, no BSEP inhibition), hepatic failure with palbociclib was recorded in a quarter of liver reports in FAERS: these findings, together with the potential occurrence of pseudocirrhosis with liver failure recently documented in two patients (Vuppalanchi et al., 2017), strengthen the importance of pursuing monitoring and vigilance in the real-world post-marketing setting.

Dedicated pharmacovigilance studies should be designed to better characterize the reporting pattern of idiosyncratic DILI, accounting for major bias (e.g., disproportionality by therapeutic area, case-by-case assessment, and adjustment for time on the market, drug use, concomitant hepatotoxic drugs) and correlate the observed reporting frequency with binding affinities (e.g., CDK4/6, CDK2) and all pharmacokinetic parameters (e.g., volume of distribution) to actually verify the putative relationship between the risk (reporting) of liver damage and pharmacodynamics/pharmacokinetics. We believe that this approach (critical analysis of the literature pertaining to pharmacological features implicated in DILI onset) can be extended to other promising oral anticancer drugs for breast cancer (e.g., P13K-alpha inhibitors) to timely identify DILI signals and early intercept specific drug signatures.

## Author Contributions

ER and FP contributed to the conception and design of the review. ER collected and analyzed the data, and provided the first draft of the manuscript. FP revised the manuscript for important intellectual content. Both authors contributed to final manuscript revision, and read and approved the submitted version.

## Funding

No sources of funding were used to assist in the preparation of this study. The authors are supported by Institutional Research Funds of the University of Bologna (Ricerca Fondamentale Orientata).

## Conflict of Interest

ER reports personal fees from Novartis (consultancy for drug-induced liver injury). The remaining author declares that the research was conducted in the absence of any commercial or financial relationships that could be construed as a potential conflict of interest.
